# Clinical inertia in asthma

**DOI:** 10.1038/s41533-023-00356-5

**Published:** 2023-10-14

**Authors:** Yosuke Fukuda, Tetsuya Homma, Hironori Sagara

**Affiliations:** 1 Department of Medicine, Division of Respiratory Medicine, Yamanashi Red Cross Hospital, 6663-1 Funatsu, Fujikawaguchiko-machi, Yamanashi Japan; 2 Department of Medicine, Division of Respiratory Medicine and Allergology, Showa University School of Medicine, 1-5-8 Hatanodai, Shinagawa-ku, Tokyo Japan

**Keywords:** Asthma, Patient education, Respiratory signs and symptoms

## Abstract

Despite advances in pharmaceutical treatment in recent years, a relatively high proportion of patients with asthma do not have adequate asthma control, causing chronic disability, poor quality of life, and multiple emergency department visits and hospitalizations. A multifaceted approach is needed to overcome the problems with managing asthma, and clinical inertia (CI) is a crucial concept to assist with this approach. It divides clinical inertia into three main categories, which include healthcare provider-related, patient-related, and healthcare system-related CI. The strategies to overcome these CI are complex, and the M-GAP approach, which combines a multidisciplinary approach, dissemination of guidelines, utilization of applications, and development and promotion of low-cost prescriptions, will help clinicians.

## Current situation regarding care for asthma

Asthma is a chronic respiratory disease characterized by chronic airway inflammation with symptoms such as wheezing, cough, sputum production, dyspnea, and chest pain, which cause a disease burden and loss of healthy life. In 2019, 262 million people worldwide of all ages were affected by asthma (https://www.who.int/news-room/fact-sheets/detail/asthma)^1^. The two goals of asthma treatment are achieving good control and maintaining activity and avoiding future risks such as asthma-related death, exacerbations, and adverse drug reactions (https://ginasthma.org/gina-reports/)^2^. Pharmacotherapy, directed at treating chronic airway inflammation, is an essential tool for achieving these two goals. Inhaled corticosteroids (ICS), which became the mainstay of treatment in the 1990s, significantly improved symptom control, resulting in a significant reduction in the number of patients dying from asthma. From 1990 to 2015, the age-adjusted mortality rate decreased by 58.8% worldwide^3^, and from 2010 to 2019, the mortality rate per 100,000 population decreased by 17.4%^4^. Furthermore, the development of biological agents has resulted in better disease control and can be of benefit for providing personalized medicine to patients with asthma. For example, omalizumab, an anti-IgE antibody, can reduce respiratory symptoms^5^ and unscheduled hospital visits and hospitalizations in patients with asthma^6,7^. Similarly, mepolizumab^8^, benralizumab^9^, and reslizumab^10,11^, which target interleukin 5 (IL-5) signaling; dupilmab^12^, which targets IL-4 signaling; and tezepelumab^13,14^, which targets thymic stromal lymphopoietin (TSLP) signaling, are effective at reducing the number of emergency room visits and hospitalizations due to acute exacerbations, improve clinical symptoms and respiratory function, and reduce oral corticosteroid use. These medications are essential for maintaining the quality of life, especially for patients who have refractory asthma.

However, advances in pharmacotherapy do not necessarily achieve good control of asthma and avoidance of future risks. For example, the European Community Respiratory Health Survey (ECRHS), a Europe-wide multicenter study, found that 85% of patients with asthma who had used ICS in the previous year had asthma that was partially controlled or uncontrolled^15^. Moreover, 39% of patients with uncontrolled asthma and 53% of patients with partially controlled asthma had a treatment status equivalent to Global Initiative for Asthma (GINA) treatment step 1 (not using antiasthma medications daily)^15^. A study based on the Japanese health insurance claims database found that 32% of patients with severe asthma and 16% of patients with mild-to-moderate asthma, as defined by international guidelines, had uncontrolled disease^16^. High age-standardized disability-adjusted life-years (DALYs) have been reported, not only in countries with a low-to-medium socio-demographic index (SDI), such as Southeast Asia and Africa but also in countries with a high SDI, such as the United States and parts of Europe^4^. Furthermore, another study found that few patients with severe asthma had well-controlled asthma, based on the years lived with a disability, one of the components of DALYs^4^. These findings suggest that the prevalence of inadequately controlled asthma is high and that multiple preventable risks are not sufficiently avoided, contributing to disability and death from asthma.

## Clinical inertia

### What is clinical inertia?

A multifaceted approach is needed to overcome the problems with managing asthma, and clinical inertia (CI) is a crucial concept to assist with this approach. The concept of CI was proposed by Phillips et al.^17^ in 2001 and has been applied mainly to lifestyle-related diseases such as hypertension, dyslipidemia, and diabetes. Phillips et al.^17^ defined CI as the failure of healthcare providers to initiate or intensify appropriate treatment despite various therapeutic advances having clarified treatment goals. In an observational study of approximately 1500 patients with hypertension treated by 500 physicians, 30% of the participants had CI that needed to be corrected^18^. Even when telemonitoring triggered an intervention alert that the average home blood pressure was elevated over 2 weeks, more than half of the physicians reported that they did not intensify treatment because they judged the blood pressure to be within an acceptable range^19^. This trend in hypertension is also true for dyslipidemia and type 2 diabetes mellitus. For example, a survey found that 86% of physicians thought that CI was a problem in the management of dyslipidemia^20^. A Spanish study of patients with type 2 diabetes found that CI was present in one-fifth to one-quarter of patients and that the prevalence varied according to the patient’s hemoglobin A1c level^21^. Okonofua et al.^22^ defined this condition as therapeutic inertia (TI) and used it to stratify patients. They found that in patients with hypertension, a higher TI score was correlated with poorer blood pressure control. Multivariate analysis also showed a significant negative correlation between TI and blood pressure control^22^. In patients with lifestyle-related diseases, such as treatable cardiovascular risk factors, these factors affect the clinical outcomes. CI is a significant problem that needs to be overcome by patients, their families, and healthcare providers for patients to maintain their quality of life and lead healthy lives.

## Clinical inertia in asthma

**Fig. 1:**
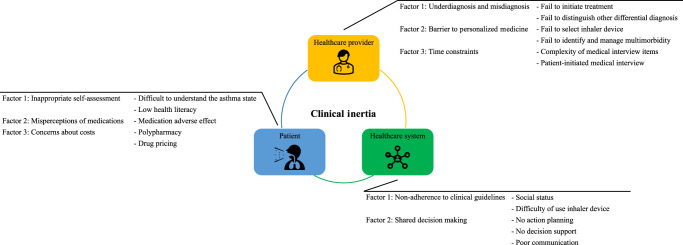
Clinical inertia associated with asthma clinical practice. CIs include healthcare-related, patient-related, and healthcare system-related. Each CI consists of multiple elements. CI clinical inertia.

CI is also present in asthma practice. In some cases, asthma is not controlled, yet appropriate therapeutic intervention or intensification is not provided, or treatment that is no longer needed is continued without reducing the dose or discontinuation. One study found that 39% of patients diagnosed with “definite asthma” were not receiving any medication^23^. Another study found that 39% of patients with uncontrolled asthma and 53% of patients with partially controlled asthma were not using daily medication for asthma control, equivalent to GINA treatment step 1^15^. CI is not necessarily caused by a single factor but is due to a complex interplay of factors and needs to be addressed using a multifaceted approach. Factors affecting CI in asthma fall into three main categories: those related to the healthcare provider (contribution rate: 50%), those related to the patient (contribution rate: 30%), and those related to the healthcare system (contribution rate: 20%), although some factors are shared between categories^24^ (Fig. 1).

## Healthcare provider-related clinical inertia in asthma

### Under- and misdiagnosis in asthma

Asthma is one of those diseases for which diagnostic criteria are not absolute. The GINA guidelines suggest a flow for diagnosing asthma if respiratory symptoms typical of asthma, such as wheezing, shortness of breath, chest tightness, and cough, are present. Past medical history and laboratory results are useful for the diagnosis of asthma, and spirometry tests show expiratory airflow obstruction^2^. The proposed flow is to diagnose asthma when there is reversibility or diurnal variation in peak flow.

Management of patients with asthma requires that healthcare providers take two steps: diagnosis and treatment. One form of CI is underdiagnosis, resulting in the patient not being initiated on appropriate treatment. In a study of adult patients with morbid obesity (body mass index: ≥35 kg/m^2^), of the 54 patients whose physicians ruled out a diagnosis of asthma, 17 (31%) were later determined to have undiagnosed asthma based on respiratory symptoms suggesting airflow limitation using the Asthma Quality of Life Questionnaire (AQLQ), Asthma Control Questionnaire (ACQ), and spirometry^25^. Adams et al.^26^ examined 3422 randomly selected adults aged 18 years and older, who were examined by a physician, tested for allergies, and underwent spirometry. They found that 11.6% of participants had asthma but that asthma was undiagnosed in 19.2% of those with asthma. Participants with undiagnosed asthma had lower values for respiratory function, such as forced expiratory volume in one second and forced vital capacity, compared with those with diagnosed asthma.

An analysis of spirometry patterns showed that 6.1% of patients diagnosed with asthma had chronic obstructive pulmonary disease (COPD), and conversely, 56.3% of patients diagnosed with COPD were re-diagnosed with asthma. In other words, some healthcare providers do not clearly distinguish between asthma and COPD^27^. A study of patients with COPD and no indication for ICS use found that one-quarter of the patients had a history of ICS prescription^28^.

This underdiagnosis and misdiagnosis due to unawareness of asthma are crucial components of CI among healthcare providers, which hinders the introduction of appropriate initial and intensified treatment.

### Barriers to personalized medicine

The objectives of treatment in patients with asthma are to achieve good disease control, maintain activity levels, and minimize the risk of asthma-related death, exacerbations, persistent airflow limitation, and side effects^2^. Thus, patients need to receive appropriate individualized treatment based on their complex personal background and the severity of the disease. However, many healthcare providers do not take all the relevant factors into consideration and make inappropriate choices of treatment methods.

One of these issues is related to inhalation techniques. According to previous reports, almost three-quarters of patients with asthma have an imperfect inhalation technique^29,30^. In a survey of Dutch residents, 29.5% of patients treated equivalent to GINA steps 4–5 required high-dose oral corticosteroids, of whom 78% had problems with inhalation technique or medication adherence^31^, suggesting the possibility of inappropriate inhalation device selection by healthcare providers.

Multimorbidity, with asthma as the index disease at the center of care, is another barrier to personalized medicine. Asthma can be comorbid with other conditions such as sinusitis, COPD, obstructive sleep apnea, hyperventilation syndrome, gastroesophageal reflux disease, eosinophilic polyangiitis with granulomatosis, vocal cord dysfunction, obesity, heart failure, pregnancy, endocrine disorders, psychological disorders. In addition, 40% of asthma patients had problems with their cardiovascular system, and more than half had problems with their gastrointestinal system in UK primary care data^32^. Indeed, cluster analysis in the Asthma-E3N study showed that asthma prognosis was poorer in clusters that included asthma groups with problems in these systems^33^. Because these can be “treatable traits,” healthcare providers need to consider limited medical resources and complex social contexts and tailor a comprehensive approach to individual patients.

Healthcare providers may prescribe routine medications without adjusting prescriptions to individual patient characteristics, and many healthcare providers adopt reactive rather than proactive treatment strategies. By setting appropriate goals based on the patient’s condition, healthcare providers can proactively coordinate initial and maintenance treatment to achieve asthma treatment goals.

### Time constraints

One of the causes of healthcare provider-related CI is the issue of time constraints in practice. For example, in asthma practice, limited time is spent on patient education, primarily in medical interviews and inhalation techniques. Asthma is one of the diseases with a large environmental component; therefore, sufficient time must be allocated to conduct medical interviews. In addition, better patient education is important for improving asthma control^34–37^. Although practice hours have gradually increased in the United States and the United Kingdom since the 1990s^38^, more than half of the respiratory specialists questioned said that when diagnosing patients with occupational asthma, they did not have adequate time with the patient^39^. Similar findings have been reported by family physicians^40^. One possible explanation is that more time than necessary is devoted to patient-initiated medical interviews, causing time constraints in practice.

Listening to patient complaints is an important skill for healthcare providers, but it is also important to use time with patients efficiently. These findings suggest that clinic time in asthma care is not always sufficient and that this is linked to CI.

## Patient-related clinical inertia in asthma

### Inappropriate self-assessment of asthma control

Strengthening patients’ health literacy is one of the most critical challenges. The largest obstacle is that many patients do not recognize that their asthma is poorly controlled. Some patients deny even having asthma. In other words, patients lack awareness that poor asthma control reduces their quality of life and increases the frequency of unscheduled emergency department visits and hospitalizations required due to asthma exacerbations. Since ancient times, it has been reported that there has been a perception gap between healthcare providers and patients regarding asthma control. An online survey of 8000 patients with asthma in 11 European countries found that 62% of patients who could be considered to have uncontrolled asthma, according to the GINA guidelines, perceived themselves as healthier than their peers, and 57% of patients perceived their symptoms as less severe; 35 and 26% of patients whose asthma was partially controlled or uncontrolled used only symptomatic treatment as needed^41^. The same is true in low-income countries. An observational study conducted in a large hospital in Ethiopia found that among patients whom physicians judged to be partially controlled or uncontrolled according to GINA guidelines, only 21% of the patients themselves were aware that their asthma was poorly controlled^42^. In a U.S. study of patients with asthma and primary care physicians, more than 80% of patients who discontinued controller medications did so without medical supervision^43^.

These findings suggest that self-assessment of asthma control by patients tends to be less rigorous than that of healthcare providers^44^. Although healthcare providers perceive the prevention of exacerbations and emergency room visits as important treatment goals, patients perceive the ability to perform daily activities as important^45^, and differences in perceptions between patients and healthcare providers regarding asthma treatment goals and limiting future risk may lead to CI.

### Misperceptions about asthma medications

The second factor contributing to patient-relative CI is the patient’s perception of asthma medications. This is primarily related to avoidance and misperception of medication side effects and adverse effects. Concerns about medication side effects tend to be greater in patients with severe asthma^46^, with 18–60% of patients expressing an aversion to ICS, a key medication in the stable phase of asthma treatment^47–50^. In an observational study of asthma in Canadian adults, 36% of patients misperceived ICS as less effective with long-term use and were concerned about side effects such as weight gain, infection, bone fragility, and growth disturbance^49^. Many of the patients not using ICS stated that they used ICS only when necessary or did not want to use ICS when they were asymptomatic^50^. Decreased use of ICS may lead to excessive use of short-acting beta-agonists (SABAs), which in turn may increase the risk of asthma exacerbations^51^.

### Concern about costs associated with asthma care

The third factor contributing to patient-related CI is concern about the cost of healthcare. A cross-sectional survey conducted in Australia found that 52.9% of adult patients with asthma and 34.3% of parents with asthmatic children were withholding medication due to cost. This included patients who used smaller doses or skipped doses of asthma medications to make them last longer^52^. This study found that among adults with asthma, patients who were younger, male, and who reported having concerns about their medications and difficulty discussing medication changes with their physicians were more likely to refrain from using medications due to cost-related concerns^52^. Another study found that 10.2% of patients used a fixed-dose combination of an ICS and long-acting bronchodilator that needed to be inhaled twice daily, only once daily, to save money^53^.

Among asthma medications, biologics are among the most expensive treatment options, and the cost of treatment is often a barrier to their use. A study conducted in the United States to investigate adherence to biologics in biologic-naïve patients with moderate to severe asthma found that 73% received the medication only in the clinic, and 20% self-injected the medication at home^54^. In the group of patients receiving clinic-based dosing, adherence decreased by 2% for each $1000 increase in cost to the patient^54^. Moreover, in patients with conditions other than asthma, polypharmacy may be closely related to cost^54^. These results suggest that reducing the financial burden on patients is essential in maintaining medication adherence and preventing patient-related CI.

## Healthcare system-related clinical inertia in asthma

### Non-adherence to clinical practice guidelines

The GINA is an international guideline for asthma care that is updated annually^2^, and guidelines for asthma have been developed in many countries. Treatment in accordance with these asthma guidelines is expected to improve clinical outcomes. A cross-sectional study conducted in Italy showed that adherence to GINA guidelines by general internists was associated with better asthma control^55^. In addition, following the GINA guidelines and using adequate doses of anti-inflammatory medications, including ICS, enabled control to be achieved, especially in patients with mild or moderate asthma^56^.

However, compliance with asthma guidelines is not high: In a study of 5107 patients with asthma who participated in the Nurses’ Health Study^57^, only 57% of those with mild persistent asthma and only 32% with severe asthma^58^. Family physicians and internists were significantly less likely than pediatricians to adhere to the guidelines (71.6% vs. 50.6%)^59^. A study among patients attending pulmonary disease or allergy clinics found that more than half the patients had poorly controlled disease and that among those with poorly controlled disease, 40% were eligible for triple ICS, long-acting β2-agonist, and long-acting muscarinic antagonist (ICS/LABA/LAMA) therapy, and 20% were eligible for biologic agents^60^. Reasons for the lack of appropriate guideline-compliant treatment are multifactorial and include asthma severity, advanced age, number of comorbidities, low socioeconomic status, difficulty using inhaled medications, and whether the healthcare provider was an asthma specialist^58,61–63^.

Considering the above, the multifactorial issues related to guideline adherence need to be addressed in order to resolve CI in asthma and to achieve better asthma control.

## Lack of shared decision-making by multiple professions

Although healthcare professionals should actively participate in shared decision-making to ensure the quality of life of patients with asthma^64^, some patients find that consulting healthcare professionals on matters related to asthma control can be laborious and futile. One study found that 78% of patients with poorly controlled severe asthma had consulted healthcare professionals about asthma control in the previous year but that patients’ opinions about treatment were considered in less than one-third of patients^65^. Other studies have found that only 20% of patients had an action plan developed by a physician or nurse^66^ and that 70% of patients with asthma believed that they were responsible for managing their asthma^67^. Some of this lack of shared decision-making may be partially due to a lack of communication not only between patients and healthcare providers but also among healthcare providers. Shared decision-making needs to be addressed through a comprehensive approach that includes physicians, nurses, pharmacists, and healthcare managers.

## Overcoming clinical inertia in asthma

Although the multifactorial nature of CI in asthma care is not easy to solve, several potential solutions exist. First, a comprehensive approach to patients is needed not only by physicians but also by pharmacists, nurses, and other healthcare professionals. Physicians can perform only a limited number of tasks, and a multidisciplinary approach is fundamental, especially for overcoming CI, due to factors such as shared decision-making and time constraints. For example, in a study conducted on patients with maternal asthma, patient education, including inhalation instruction and the use of an electronic spirometer by a pharmacist, resulted in a significant reduction in ACQ scores in the intervention group compared with a control group^68^. Similarly, an Australian study of a pharmacist-led pharmacy asthma service intervention for patients with poorly controlled asthma that addressed adherence, inhalation techniques, and allergic rhinitis showed that asthma treatment was reviewed in approximately half of the patients who consulted a general practitioner based on the pharmacist’s suggestion^69^. Other studies have found that nurse-led interventions by so-called “asthma nurses” increased knowledge of inhaled medications^70^ and decreased the rate of acute exacerbations^71^. Based on these findings, it is assumed that healthcare providers other than physicians, such as nurses and pharmacists, can directly contribute to asthma care by providing lifestyle guidance, including the accuracy of inhaler technique and environmental maintenance, through online medical care and home care medicine in clinical practice. They can also play a “hub” role by providing feedback to physicians on patient-related issues they have identified through their approaches, which may provide clues for multidisciplinary problem-solving and modification for better management.

As noted above (see Section “Non-adherence to clinical practice guidelines”), evidence-based guidelines have improved asthma control, but adherence rates are not always high^58^. One study found that maintenance and reliever treatment, one of the treatments recommended by GINA, was practiced in only about 15% of patients with GINA step 1–2 equivalent^72^. In our opinion, there are two main reasons why adherence to the guidelines is not high. The first is the low awareness of the guidelines themselves, and the second is the lack of proposals for measures in different regions with different resources available for utilization. There are two main strategies to attack this problem of compliance with the guidelines: first, a publicity strategy using the media. The first is a media-based advertising strategy. In recent years, web services, including social networking services, have developed at an accelerated pace, and we believe that utilizing these services will be more helpful in disseminating the guidelines than the classic method of distributing printed materials^73^. Another measure to increase awareness of the guidelines is to create opportunities for guideline-based distance learning programs to increase familiarity with asthma care as part of the web services^74,75^. The second is to share the guidelines with the local medical community. Specifically, providing workshops for small group discussion of the guidelines in the community is useful^76^. It was reported that, compared to asthma specialists, general practitioners do not adhere to spirometry, develop asthma action plans, and repeatedly check inhalation techniques^77^, and we believe that these issues can be addressed by reviewing the guidelines from the perspective of resource utilization in the community, leading to personalized medicine.

A third strategy is the promotion of digital information using mobile health applications (apps). This fact suggests that apps may fill the gap in the knowledge of health professionals and patients about asthma and may contribute to shorter consultation times^78,79^. A randomized controlled trial examining the use of audiovisual inhaler reminders and feedback found that adherence at 6 months was significantly higher in the inhaler reminder group than in the control group^80^. Similarly, a study examining the usefulness of the Propeller Health system, an inhaler sensor, in adult patients with asthma reported that use of the Propeller Health system not only reduced the frequency of SABA use but also led to clinical improvement in patients with low Asthma Control Test (ACT) scores^80,81^. Smoking is an avoidable exposure factor, yet one study found that 13–35% of patients with asthma were current smokers^82^. Treatment combined with smartphone apps to assist in smoking cessation has been reported to be useful^83^. Asthma-related apps can be useful, but one problem with using the apps is that patients and physicians differ in the functions they require of the apps. Specifically, over 90% of patients with asthma wanted a mobile app with asthma education materials, symptom prediction, and action plan features, whereas asthma specialists preferred an app with symptom scores and information about air pollution^84^. Furthermore, it is unclear who should use the app, and issues regarding security and language have not been resolved^85^. Although various issues remain to apply and deploy these apps in the real world, apps for asthma may increase patients’ health literacy and understanding of the disease. It can also contribute to the selection of inhaler devices and the creation of action plans.

Fourth, it is important to promote the development and use of lower-cost medications. As noted above (see Section “Concern about costs associated with asthma care”), cost is a major burden for patients, their families, and healthcare institutions, and reducing this burden is an important aspect of national policy. For example, generic medications and biosimilars to salmeterol/fluticasone generics have been shown to improve ACT scores and respiratory function^86^, and their clinical benefit in the short term is similar to that of the original medication^87^. A biosimilar of omalizumab has been shown to be non-inferior to the existing product in terms of clinical efficacy and toxicity^88,89^. This approach to medications can remove psychological constraints on medication safety and cost for patients and healthcare providers, and the widespread availability of medications is likely to promote a comprehensive approach by the healthcare team.

**Fig. 2:**
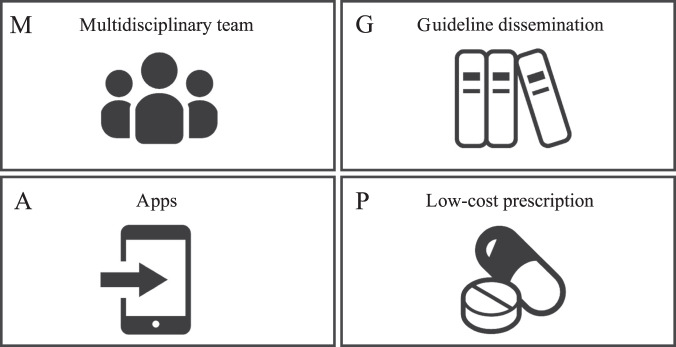
An approach to addressing clinical inertia in asthma care. We propose the promotion of an M-GAP approach: a Multidisciplinary approach, dissemination of Guidelines, utilization of Applications, and promotion of low-cost Prescriptions. Apps applications.

Based on these considerations, we propose these four approaches (multidisciplinary approach, dissemination of guidelines, utilization of applications, and development and promotion of low-cost prescriptions) as the M-GAP approach to resolving CI (Fig. 2).

## Future perspective of clinical inertia in asthma

CI applies not only to an intensification of treatment but also to follow-up of patients who are discontinuing treatment or transitioning to a lower dose, including discontinuing medications that are no longer needed^90^. Patients on multiple medications are a prime example. One study found that more than half of older patients wanted to reduce the number of medications that they were taking if their doctors deemed it possible^91^. A comparison of patients on multiple medications with those not on multiple medications showed that asthma control was predominantly worse in patients on multiple medications^33^. The same is true for asthma overdiagnosis. Aaron et al.^92^ reported that among patients diagnosed with asthma during the past 5 years, approximately 30% of patients had no evidence of asthma when reevaluated using symptom monitoring, spirometry, and peak flow. Similarly, in a study conducted in Sweden, 34% of patients diagnosed with asthma were found not to have asthma when evaluated by an allergist based on respiratory function tests and a methacholine challenge test^93^. This suggests the importance of distinguishing between pure CI and “appropriate inaction” in asthma care^94^. Therefore, it is crucial for healthcare providers to improve their practice skills by resolving CI using an M-GAP approach and for patients to improve their self-management skills.

### Reporting summary

Further information on research design is available in the Nature Research Reporting Summary linked to this article.

### Supplementary information

Reporting Summary

## Data Availability

Data sharing is not applicable to this article as no datasets were generated or analyzed during the current study.
